# Retraction Note: Aging-associated distinctive DNA methylation changes of LINE-1 retrotransposons in pure cell-free DNA from human blood

**DOI:** 10.1038/s41598-022-07275-4

**Published:** 2022-02-22

**Authors:** Wardah Mahmood, Lars Erichsen, Pauline Ott, Wolfgang A. Schulz, Johannes C. Fischer, Marcos J. Arauzo‑Bravo, Marcelo L. Bendhack, Mohamed Hassan, Simeon Santourlidis

**Affiliations:** 1 Epigenetics Core Laboratory, Institute of Transplantation Diagnostics and Cell Therapeutics, Medical Faculty, Heinrich-Heine University Duesseldorf, Moorenstr. 5, 40225 Duesseldorf, Germany; 2 Department of Urology, Medical Faculty, Heinrich-Heine University, Duesseldorf, Germany; 3 Institute of Transplantation Diagnostics and Cell Therapeutics, Medical Faculty, Heinrich-Heine University Duesseldorf, Moorenstr. 5, 40225 Duesseldorf, Germany; 4 Group of Computational Biology and Systems Biomedicine, Biodonostia Health Research Institute, 20014 San Sebastián, Spain; 5 IKERBASQUE, Basque Foundation for Science, 48009 Bilbao, Spain; 6 Department of Urology, University Hospital, Positivo University, Curitiba, Brazil; 7 Department of Surgery, Tulane University School of Medicine, New Orleans, LA 70112 USA; 8 Institut National de la Santé et de la Recherche Médicale, University of Strasbourg, 67000 Strasbourg, France

Retraction of: *Scientific Reports* 10.1038/s41598-020-79126-z, published online 17 December 2020

The Authors have retracted this article.

After publication concerns were raised about duplication of lanes within Figure 2 and between Figure 2 and Figure 5. The Authors were no longer able to locate the original data underlying these figures.

Lars Erichsen, Pauline Ott, Wolfgang A. Schulz, Johannes C. Fischer, Marcos J. Arauzo-Bravo, Marcelo L. Bendhack, Mohamed Hassan and Simeon Santourlidis agree to the retraction and its wording. Wardah Mahmood has not responded to correspondence from the Editors about this retraction.

